# Transcutaneous Electrical Nerve Stimulation (TENS) Versus Microcurrent Electrical Neuromuscular Stimulation (MENS) Combined with Kinesiotherapy in Partial-Thickness Rotator Cuff Tears: A 12-Month Randomized Controlled Trial

**DOI:** 10.3390/healthcare14193290

**Published:** 2026-10-02

**Authors:** Sotiria Vrouva, Konstantinos Chanopoulos, Eftychia Andreou, Magdalini Stamou, George A. Koumantakis

**Affiliations:** 1 Physiotherapy Department, School of Health and Care Sciences, University of West Attica (UNIWA), 12243 Athens, Greece; mscphys25002@uniwa.gr (E.A.); mstamou@uniwa.gr (M.S.); gkoumantakis@uniwa.gr (G.A.K.); 2 Department of Application Developments, Hellenic Military Geographical Service (HMGS), 11362 Athens, Greece; k_hanos@yahoo.com

**Keywords:** partial-thickness rotator cuff tears, Microcurrent Electrical Neuromuscular Stimulation (MENS), Transcutaneous Electrical Nerve Stimulation (TENS), kinesiotherapy, longitudinal follow-up, clinico-radiological paradox, Minimal Clinically Important Difference (MCID)

## Abstract

**Background/Objective:** Partial-thickness rotator cuff tears represent a distinct, highly prevalent clinical entity governed by a multifactorial continuum of intrinsic senescence and extrinsic biomechanical overload, rather than an oversimplified acute traumatic versus degenerative dichotomy. While conservative management centered on kinesiotherapy is primary, Transcutaneous Electrical Nerve Stimulation (TENS) and Microcurrent Electrical Neuromuscular Stimulation (MENS) are widely utilized as adjunctive electrophysical modalities. However, empirical trials directly comparing their long-term therapeutic longevity and structural stability remain scarce. This prospective, randomized, participant- and outcome assessor-blinded clinical trial evaluated the 12-month effectiveness of combining structured kinesiotherapy with either TENS or MENS in patients with partial-thickness rotator cuff tears. **Methods:** Of 47 candidates screened via standardized physical provocation tests and musculoskeletal ultrasound, 5 were excluded prior to treatment allocation. Exactly 42 eligible patients (*n* = 42; 22 males, 20 females; mean age: 46.71 ± 12.24 years) were formally enrolled and randomized (1:1) to receive 15 supervised sessions of either conventional sensory TENS (*n* = 21; 100 Hz, 10–15 mA) or subsensory MENS (*n* = 21; 50 Hz, 100 μA) alongside an identical kinesiotherapy program. Longitudinal evaluations were conducted at baseline, 1, 3, 6, and 12 months post-treatment, achieving a 100% participant retention rate (*n* = 42). Clinical outcomes comprised the Shoulder Pain and Disability Index (SPADI), an 11-point Numeric Rating Scale (NRS) for pain, and the EuroQoL 5-Dimension Questionnaire (EQ-5D). Structural tear dimensions were monitored via serial ultrasonography. Longitudinal data were analyzed using two-way repeated-measures Analysis of Variance (RM-ANOVA)with Bonferroni adjustment applied for post hoc pairwise comparisons for normally distributed metrics and the Friedman and Mann–Whitney *U* tests for non-parametric variables, assessing both within-group trajectories and between-group interaction effects across time. **Results:** Baseline demographic, occupational, and clinical characteristics showed no significant differences between cohorts (*p* > 0.05). At the 3-month post-treatment mark, both groups exhibited equivalent, statistically significant within-group improvements across all clinical scores (*p* < 0.01). However, longitudinal trajectories diverged significantly thereafter. Between-group comparative analyses established that the MENS cohort achieved statistically and clinically superior outcomes over the TENS cohort at both 6 and 12 months. Specifically, at the 12-month primary endpoint, between-group comparisons favored MENS for SPADI Pain (mean difference: 14.39 ± 3.51, 95% CI: 7.28 to 21.50, *p* < 0.001), SPADI Disability (mean difference: 13.56 ± 2.89, 95% CI: 7.71 to 19.42, *p* < 0.001), NRS pain (mean difference: 2.33 ± 0.32, *p* < 0.001), and EQ-5D health status (*p* < 0.001), substantially exceeding established Minimal Clinically Important Difference (MCID) benchmarks. Musculoskeletal ultrasound demonstrated post-intervention stabilization of tear progression in the MENS cohort (0.02 mm secondary depth increase between months 3 and 12), whereas the TENS arm exhibited progressive expansion (0.45 mm increase), presenting a classic clinicoradiological paradox against functional recovery. **Conclusions:** Both TENS and MENS serve as effective adjunctive modalities for acute symptom alleviation when paired with kinesiotherapy. However, based on robust between-group comparisons, subsensory MENS provides significantly superior long-term clinical durability, tear containment, and functional stability over a 12-month timeline, making it the preferred adjunctive electrophysical intervention for sustained conservative management.

## 1. Introduction

Rotator cuff tears represent one of the most prevalent musculoskeletal pathologies affecting the shoulder complex, exhibiting a particularly high incidence among middle-aged and older populations [1,2,3]. Because the rotator cuff is structurally comprised of four distinct musculotendinous units—the supraspinatus, infraspinatus, subscapularis, and teres minor—structural disruptions may manifest as an isolated tendinous lesion or progress to involve adjacent tendons simultaneously [1,2].

Rather than adhering to an oversimplified dichotomy between isolated acute trauma and insidious degeneration, contemporary pathophysiological frameworks conceptualize the etiology of rotator cuff tears—and specifically partial-thickness tears—as an intricate, multifactorial continuum [4,5]. Pathogenesis is governed by a dynamic interplay between intrinsic biological senescence and extrinsic biomechanical stressors [5,6]. Intrinsic vulnerabilities encompass age-dependent hypovascularity within the insertional “critical zone,” tenocyte metabolic dysfunction and apoptosis, and the progressive disorganization of the type I collagen extracellular matrix [4,7]. Concurrently, extrinsic factors—including morphological variations of the coracoacromial arch, dynamic subacromial or internal impingement, internal rotation deficits, and cumulative tensile/shear stresses—generate localized stress concentrations across the tendon footprint [5,7,8]. Consequently, even when an acute macrotraumatic onset is reported clinically, it predominantly reflects an acute-on-chronic structural failure superimposed upon pre-existing, subclinical microstructural tissue degeneration [6,9,10].

Pathoanatomically, partial-thickness tears exhibit distinct spatial distributions, occurring across the myotendinous junction, the articular or bursal surfaces, within the substance of the tendon itself (intratendinous lesions), or as complex multiplanar disruptions [1,7].

Clinically, the symptomatic presentation of partial-thickness tears is characterized by marked heterogeneity [7,8]. Although patients frequently report deep, persistent shoulder pain that typically exacerbates at night, the associated impact on joint kinematics varies considerably across individuals [5,7,11]. Some individuals develop secondary capsular tightness or pain-induced muscle guarding, leading to measurable motion deficits—most notably restricting glenohumeral internal rotation or terminal elevation [1,12]. Conversely, a substantial subset of patients retains full, pain-free passive range of motion, experiencing symptoms exclusively during resisted contractions, end-range active elevation, or high-demand overhead tasks [5,8]. This clinical divergence underscores that pain-mediated motor inhibition and load intolerance during functional activities—rather than obligate passive capsular contracture—serve as the primary drivers prompting patients to seek orthopedic care [8,12].

Initial clinical evaluation relies heavily on thorough patient history and physical examination, routinely incorporating provocative orthopedic maneuvers such as the Neer impingement sign, the Hawkins-Kennedy test, and the Jobe (empty can) test [7,13]. Crucially, however, these clinical tests demonstrate high sensitivity but notoriously low diagnostic specificity for isolated rotator cuff tears [13,14]. Rather than functioning as pathognomonic indicators of tendinous disruption, they serve as non-specific symptom-provocation maneuvers reflecting generalized subacromial pain syndrome (SAPS), subacromial bursitis, or regional tissue irritability [8,13]. As such, provocative maneuvers cannot reliably differentiate partial-thickness tears from intact, inflamed tendons or other subacromial pathologies [8,14]. Definitive diagnostic confirmation and anatomical staging therefore require advanced imaging, routinely accomplished via musculoskeletal ultrasonography or magnetic resonance imaging (MRI or MR arthrography) [7,10].

Within conservative management paradigms, physical therapy represents a cornerstone intervention alongside standard pharmacological management [12,15]. Rehabilitation initially focuses on symptom modulation and pain relief through diverse electrophysical modalities, including therapeutic ultrasound, diathermy, low-level laser therapy, and analgesic currents such as Transcutaneous Electrical Nerve Stimulation (TENS) and Microcurrent Electrical Neuromuscular Stimulation (MENS) [7,16]. Concurrently, structured kinesiotherapy regimens are implemented to restore glenohumeral range of motion, correct scapular dyskinesia, and facilitate targeted strengthening of dynamic stabilizers [7].

Kinesiotherapy—defined as the systematic prescription of graded, purposeful therapeutic exercise—exerts its clinical efficacy primarily through mechanotransductive pathways [17]. At the cellular level, tailored mechanical loading stimulates tenocyte metabolic activity, enhancing extracellular matrix realignment and collagen synthesis while attenuating pro-inflammatory signaling cascades [8]. Biomechanically, targeted exercise restores the dynamic force couples of the glenohumeral and scapulothoracic complexes, counteracting superior humeral head migration, decompressing the subacromial space, and fostering functional tendon load tolerance [18,19].

From a neurophysiological perspective, the therapeutic convergence of kinesiotherapy and electrotherapy modulates nociceptive transmission at both spinal and supraspinal levels, consistent with the gate control theory of pain [20,21]. Conventional TENS selectively excites large-diameter, myelinated *Aβ* afferents, thereby driving segmental presynaptic inhibition of nociceptive *Aδ* and *C* fiber inputs within the substantia gelatinosa of the spinal dorsal horn [21,22]. In contrast, MENS delivers subsensory, micro-amperage stimulation that bypasses peripheral nerve depolarization to enhance intracellular adenosine triphosphate (ATP) resynthesis, restore cell membrane homeostasis, and attenuate local algesic mediators [21,23,24]. Synchronously, kinesiotherapy reinforces segmental gating via continuous articular mechanoreceptor activation while engaging descending endogenous opioid and serotonergic inhibitory pathways, creating optimal conditions for active functional restoration [21,25].

In the current literature, direct comparative trials evaluating TENS versus MENS in rotator cuff pathologies remain exceedingly scarce [26,27]. Existing evidence is predominantly constrained to short-term observational windows—rarely exceeding three months—where both modalities frequently display comparable symptomatic efficacy in pain relief and functional scores [28,29]. However, this short-term equivalence masks a fundamental neurobiological divergence: conventional TENS acts primarily via transient, sensory-level neural gating without exerting anabolic or reparative effects on tendon microstructure [16,22], whereas MENS delivers subsensory microcurrents that stimulate mitochondrial ATP resynthesis, protein synthesis, and extracellular matrix remodeling [23,24].

Investigating their comparative longevity specifically in partial-thickness tears is of paramount clinical relevance; unlike full-thickness tears, partial-thickness lesions preserve structural continuity and retain an intrinsic capacity for biological remodeling, yet carry an inherent risk of progressive propagation if mechanical loading is resumed without adequate cellular recovery [5,7]. Whether early electrotherapeutic interventions confer durable clinical protection or merely temporary symptomatic masking once active therapy ceases remains an unaddressed empirical question.

To bridge this knowledge gap, the present study provides the first prospective, 12-month randomized investigation directly comparing the long-term therapeutic durability, functional trajectories, and structural sonographic tear containment of TENS versus MENS combined with standardized kinesiotherapy in partial-thickness rotator cuff tears.

Therefore, the aim of the present study was to evaluate the long-term effectiveness of combining conservative physical therapy—specifically comparing TENS and MENS protocols—with structured kinesiotherapy in patients diagnosed with partial-thickness rotator cuff tears, evaluated at a one-year (12-month) post-treatment follow-up.

## 2. Materials and Methods

This investigation was conducted as a prospective, randomized, participant- and outcome assessor-blinded controlled clinical trial [30,31,32]. The study protocol was approved by the Ethics Committee of the General University Hospital of Attikon (Protocol code: 3(4)/11-2-2015) and registered prospectively with ClinicalTrials.gov (Identifier: NCT03781349; registration date: 18 December 2018). The entire trial spanned approximately two years, commencing in 2018. Prior to enrollment, all participants provided written informed consent in strict accordance with the ethical principles outlined in the Declaration of Helsinki. The trial design, execution, and reporting adhered to the Consolidated Standards of Reporting Trials (CONSORT) 2010 guidelines [33].

To ensure adequate statistical power to detect clinically meaningful differences between the two intervention protocols, an a priori sample size calculation was performed using G*Power software (version 3.0.10; Heinrich Heine University Düsseldorf, Düsseldorf, Germany) [34,35]. The primary analytic model was structured as a repeated-measures Analysis of Variance (RM-ANOVA) assessing the within-subject factor (time across five evaluation intervals: baseline, 1 month, 3 months, 6 months, and 12 months), the between-subject factor (treatment cohort: MENS vs. TENS), and the interaction effect (group × time) for the primary pain SPADI Pain and functional disability (SPADI Disability) outcome measures [33,36]. The statistical input parameters were defined as: (a) a two-tailed alpha significance level (*α*) of 0.05, (b) a statistical power (1 − *β*) of ≥0.80, (c) an assumed correlation among repeated measures of 0.5, and (d) a small-to-moderate effect size (*f* = 0.22, equivalent to partial *η*^2^ ≈ 0.046) for the primary group × time interaction term [33,36,37]. The calculation established that a minimum sample of 36 participants (18 per arm) was necessary to detect the specified interaction effect reliably. To account for potential longitudinal attrition, the final recruitment cohort was established at 42 participants (21 per treatment group) [34,37].

### 2.1. Setting, Participant Screening, and Baseline Characterization

Participants were recruited from the outpatient clinical populations of two tertiary centers: the Physiotherapy Department of the 401 General Military Hospital of Athens and the Second Department of Anesthesiology Pain Unit at the General University Hospital of Attikon. Initial screening and recruitment logistics were managed by designated administrative personnel (the departmental secretary of the Physiotherapy Department, 401 General Military Hospital of Athens), who operated independently from the clinical examining and treating teams.

A total of 47 candidates were initially screened. Diagnostic eligibility required sonographic confirmation of a partial-thickness rotator cuff tear that strictly correlated with the patient’s presenting clinical symptoms [3,10]. Given the documented high prevalence of asymptomatic structural cuff tears in middle-aged and older individuals, sonographic visualization of a lesion alone was deemed insufficient for inclusion [3,38]. To confirm that the visualized lesion represented the symptomatic pain generator rather than a silent incidental radiological finding, candidates were required to demonstrate concordant clinical irritability, defined by:

Localized tenderness directly over the anatomical rotator cuff footprint during targeted sonopalpation;

Mechanical reproduction of familiar pain during active abduction within the 60° to 120° painful arc; and

Positive symptom provocation in at least two standardized provocative maneuvers (Neer impingement test, Hawkins-Kennedy test, or Jobe empty can test) [7,8,13].

Exclusion criteria comprised:

Pharmacological interventions with nonsteroidal anti-inflammatory drugs (NSAIDs) or corticosteroid injections within the four weeks preceding baseline evaluation;

Alternative pain generators, including cervical radiculopathy verified by Spurling’s test, true passive capsular restriction indicative of adhesive capsulitis, symptomatic acromioclavicular or glenohumeral osteoarthritis, and isolated calcific tendinitis [8,12];

Contraindications to electroanalgesic currents: pregnancy, open wounds or active dermatological conditions in the ipsilateral shoulder area, active or historical neoplastic disease, collagen vascular disorders, severe cardiovascular pathology (including uncontrolled arrhythmias), and implanted cardiac pacemakers or electronic devices.

Of the 47 screened candidates, 5 individuals were excluded prior to treatment commencement (failure to fulfill sonographic verification criteria, relocation, or voluntary withdrawal prior to baseline allocation). Consequently, exactly 42 participants fulfilled all eligibility criteria and were formally enrolled.

At baseline, comprehensive demographic, functional, and occupational parameters were systematically documented [5,18,39]. These included self-reported limb dominance, the symptomatic side examined, concordance with the dominant upper extremity, physical activity levels via the Tegner Activity Scale (0–10) [40], occupational physical demand (categorized into sedentary/office, light-to-moderate physical work, or heavy manual labor involving overhead lifting) [8], and regular participation in recreational overhead sports (e.g., volleyball) [7].

### 2.2. Randomization, Allocation Concealment, and Blinding Procedures

The 42 enrolled patients were randomly allocated in a 1:1 ratio to either the TENS group (*n* = 21) or the MENS group (*n* = 21) using a computer-generated electronic sequence via RandomListsTeamGenerator [33]. Allocation concealment was rigorously preserved through sequentially numbered, opaque, sealed envelopes prepared by an independent researcher uninvolved in screening or assessment. Envelopes were unsealed sequentially by an administrative assistant only after the completion of all baseline clinical and ultrasonographic evaluations.

To avoid methodological ambiguity associated with generic blinding descriptors, specific masking protocols were maintained [30,31,32,41].

**Assessor Blinding:** An independent clinical physical therapist, fully masked to group allocation, study hypotheses, and the randomization sequence, conducted all physical, clinical questionnaire, and musculoskeletal ultrasound examinations at baseline and across all longitudinal follow-up intervals [30,32].

**Participant Blinding:** Patients remained blinded to their therapeutic cohort allocation. All participants received standardized briefings describing two distinct, established therapeutic electrical currents, were treated with identical electrode layouts over the ipsilateral shoulder, and followed an identical kinesiotherapy protocol, thus preventing differential expectations or placebo disparities [16,31,41].

**Treating Therapists:** Interventions were administered by certified physical therapists who operated the electrotherapy units and supervised exercise delivery. Complete therapist blinding is unfeasible in physical therapy trials comparing milliampere sensory currents (TENS) with subsensory micro-amperage currents (MENS) due to device parameter manipulation and perceptible current feedback [16,41]. However, treating therapists were strictly separated from the diagnostic screening, clinical outcome recording, data management, and statistical analysis pipelines, and had no access to the allocation master sequence [32,41].

### 2.3. Interventional Protocols and Safety Monitoring

Both treatment arms received 15 supervised clinical sessions delivered five times weekly across three consecutive weeks. Each session combined an electrophysical modality with a standardized kinesiotherapy program administered by a single dedicated physical therapist to eliminate delivery variance.

### 2.4. Transcutaneous Electrical Nerve Stimulation (TENS) Protocol

Patients in the TENS cohort received conventional sensory TENS using a quadripolar trans-arthrodial electrode configuration flanking the deltoid and rotator cuff footprint [42,43]. Treatment was applied for 20 min per session at a continuous frequency of 100 Hz and a phase duration of 100 μs. Electrical intensity was titrated individually between 10 mA and 15 mA to elicit a distinct, strong, yet comfortable non-painful paresthesia below the motor contraction threshold [21,42].

### 2.5. Microcurrent Electrical Neuromuscular Stimulation (MENS) Protocol

Patients in the MENS cohort were treated for 24 min per session at a current frequency of 50 Hz and a subsensory intensity of 100 μA [23,24]. Stimulation was delivered through a six-electrode array adhering to manufacturer specifications: four electrodes replicated the periarticular shoulder arrangement utilized in the TENS group, while two supplemental electrodes were positioned over the ipsilateral palmar surface and the cervical sympathetic chain/stellate ganglion zone. The micro-amperage current remained strictly below the sensory depolarization threshold [23,24].

### 2.6. Standardized Kinesiotherapy and Home Exercise Program

Immediately following electrical stimulation, both cohorts engaged in an identical, structured kinesiotherapy regimen progressing across phases [18,44]:

*Phase 1 (Weeks 1–2):* Passive and active-assisted range of motion exercises, including gentle capsular stretching targeting the anterior deltoid, posterior capsule, and teres minor to relieve passive restriction without impinging the tendon footprint.

*Phase 2 (Weeks 2–3):* Closed-kinetic-chain scapular stabilization, dynamic scapulothoracic retraining, and submaximal isometric strengthening of the glenohumeral dynamic force couples.

*Phase 3 (Home Program):* A standardized home-based exercise routine consisting of 15 repetitions performed twice daily, focusing on low-load dynamic rotator cuff endurance and postural scapular retraction [18,44].

Participant safety and potential adverse events were monitored during each clinical encounter. No treatment-related adverse events, skin irritations, or dropouts occurred during the 15-session intervention period.

Participant safety and potential adverse events were systematically monitored during each clinical encounter. In accordance with safety protocols, any suspected adverse reaction or tissue injury was to be formally recorded in a standard Adverse Event Form and reported immediately to the Principal Investigator and the institutional Ethics Committee, with provisions for immediate medical evaluation and treatment cessation if necessary. No treatment-related adverse events, skin irritations, tissue burns, or protocol-related harms occurred, and no participant withdrew due to safety concerns.

### 2.7. Longitudinal Evaluation Schedule and Outcome Measures

**Figure 1:**
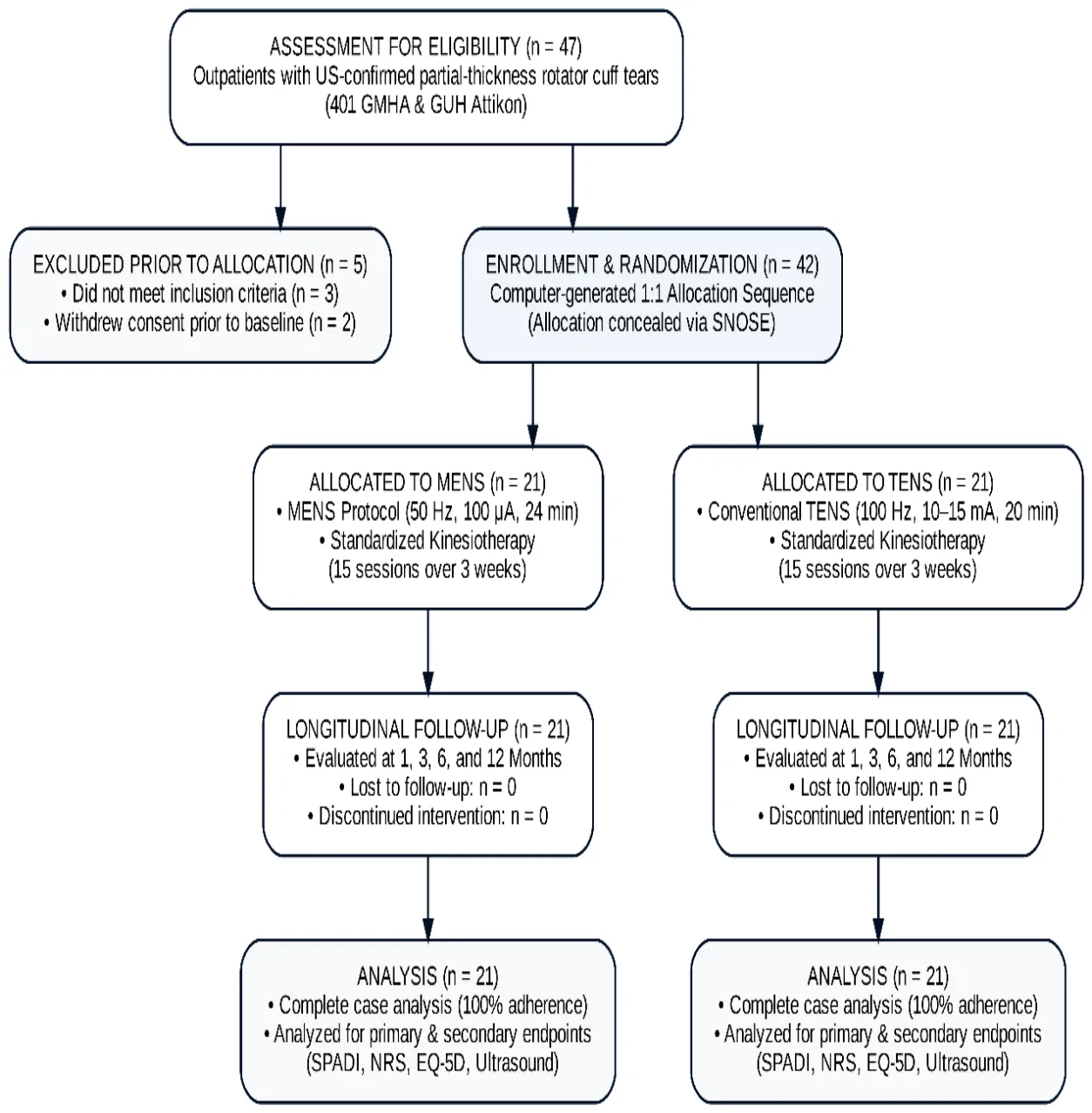
CONSORT flow diagram depicting participant screening, group allocation, 15-session treatment protocols, and follow-up evaluations across the 12-month study period.

Clinical and structural outcomes were evaluated across five standardized time points: baseline (pre-intervention, *T*_0_), 1 month (immediate post-intervention completion, *T*_1_), 3 months (*T*_2_), 6 months (*T*_3_), and 12 months (*T*_4_) post-treatment [33,45]. Patient retention was 100% (*n* = 42), with zero participant attrition or loss to follow-up through the 12-month milestone (Figure 1) [29,32].

### 2.8. Primary Clinical Outcome Measures

**Shoulder Pain and Disability Index (SPADI):** A 13-item patient-administered instrument assessing regional shoulder pain and functional disability [45,46]. It comprises two subscales: a 5-item pain subscale (0–50 points) and an 8-item disability subscale (0–80 points), aggregated into a normalized total score (0–100, where higher scores represent greater pain and disability). This study utilized the validated Greek version of the SPADI, which demonstrates excellent internal consistency (Cronbach’s *α* = 0.92 − 0.96), high test–retest reliability (ICC = 0.94), and robust construct validity in rotator cuff tears [45].

**Numeric Rating Scale (NRS) for Pain:** Pain intensity during functional movements and activities of daily living was measured using an 11-point Numeric Rating Scale ranging from 0 (“no pain”) to 10 (“worst imaginable pain”) [47,48]. The scale exhibits established validity, responsiveness, and test–retest reliability in adult musculoskeletal pain conditions [49,50,51,52,53].

**EuroQoL 5-Dimension Questionnaire (EQ-5D):** Generic health-related quality of life was quantified using the validated Greek version of the EQ-5D [54,55]. It incorporates two components: the EQ-5D health profile evaluating five dimensions (mobility, self-care, usual activities, pain/discomfort, anxiety/depression) and the EuroQoL Visual Analog Scale (EQ-VAS), a vertical rating scale from 0 (“worst imaginable health state”) to 100 (“best imaginable health state”) [54,55].

### 2.9. Musculoskeletal Ultrasound Assessment

High-resolution musculoskeletal ultrasound was performed at baseline (*T*_0_), at 3-monthfollow-up (*T*_3_) and at the final 12-month follow-up (*T*_4_) by a musculoskeletal sonographer blinded to group allocation [56]. Structural assessment evaluated tear morphology, depth, and spatial progression [56,57]. The ultrasound findings provide an additional perspective on the relationship between structural changes and clinical outcomes.

### 2.10. Statistical Analysis

Data management and statistical calculations were conducted using IBM SPSS Statistics for Windows (version 24.0; IBM Corp., Armonk, NY, USA). Continuous demographic variables were evaluated for distributional assumptions via visual inspection of Quantile-Quantile (Q-Q) plots and Shapiro–Wilk normality testing. Baseline between-group homogeneity was examined using independent-samples *t*-tests for continuous variables and Pearson’s chi-square (*χ*^2^) tests (or Fisher’s exact test) for categorical data.

For continuous and normally distributed outcomes (SPADI Pain and SPADI Disability), two-way repeated-measures Analysis of Variance (RM-ANOVA) was performed. The model specified treatment group (MENS vs. TENS) as the between-subject factor and evaluation interval (baseline, 1, 3, 6, and 12 months) as the within-subject factor, assessing the main effects of group and time as well as the primary group × time interaction effect to evaluate whether longitudinal recovery trajectories differed significantly between cohorts.

Sphericity was formally evaluated via Mauchly’s test; where the assumption of sphericity was violated, degrees of freedom were adjusted using the Greenhouse-Geisser correction. Post hoc multiple pairwise comparisons across time intervals and within groups were adjusted using the Bonferroni correction. Between-group differences at individual follow-up points were assessed via independent-samples *t*-tests.

Because the NRS pain scores and EQ-5D health state values exhibited non-normal distributions, non-parametric analytical techniques were applied. Within-group longitudinal variations across the five measurement intervals were analyzed using the Related-Samples Friedman Two-Way Analysis of Variance by Ranks. Post hoc pairwise comparisons were calculated using the Wilcoxon signed-rank test for changes within time and Mann–Whitney *U* test were performed for between-group comparisons at discrete intervals. All tests were two-tailed, and statistical significance was established at *p* < 0.05.

## 3. Results

A total of 42 patients presenting with ultrasound-confirmed partial-thickness rotator cuff tears were enrolled and completed the trial (21 randomized to the microcurrent electrical neuromuscular stimulation [MENS] cohort and 21 to the transcutaneous electrical nerve stimulation [TENS] cohort). No adverse events, tissue irritation, or protocol-related harm were reported in either group.

The overall study sample comprised 22 males and 20 females, with a mean age of 46.71 ± 12.24 years. The affected shoulder was the right side in 25 participants (59.5%) and the left side in 17 (40.5%). The dominant upper extremity was involved in 71.4% (*n* = 15) of the MENS group and 66.7% (*n* = 14) of the TENS group [18]. A single participant (2.38%) presented with bilateral symptoms; following the pre-specified protocol, intervention and outcome tracking were restricted strictly to the primary symptomatic index shoulder.

Regarding occupational demands, 17 patients (40.5%) performed sedentary or office work, 17 (40.5%) engaged in light-to-moderate physical labor, and 8 (19.0%) carried out heavy manual labor involving frequent overhead lifting [8]. Baseline activity assessments demonstrated a median Tegner Activity Scale score of 3.0 (IQR: 2.0–4.0) in both treatment arms [40], with 11 participants (26.2%) regularly participating in recreational overhead sports [7,39].

Chi-square and independent-samples *t*-tests confirmed baseline homogeneity across both cohorts, with no statistically significant differences regarding age, sex, limb dominance, affected side, occupational load, or baseline physical activity levels (*p* > 0.05) (Table 1).

### 3.1. Baseline Participant Characteristics and Group Comparability

Independent-samples *t*-tests and Pearson’s chi-square (*χ*^2^) analyses confirmed robust baseline equivalence between the two cohorts across all demographic and clinical dimensions, including mean age (*p* = 0.344), body weight (*p* = 0.312), sex distribution (*p* = 0.217), affected shoulder side (*p* = 0.341), limb dominance concordance (*p* = 0.741), occupational physical load (*p* = 0.942), Tegner activity scores (*p* = 0.884), and structural tear severity stratified by the Ellman classification (*p* = 0.293) (Table 1). Furthermore, baseline assessments confirmed no statistically significant differences between groups for any of the primary or secondary outcome measures: SPADI Pain (*p* = 0.295), SPADI Disability (*p* = 0.690), NRS pain (*p* = 0.505), and EQ-5D health status (*p* = 0.961) [29].

### 3.2. Primary Outcome Measures: Shoulder Pain and Disability Index (SPADI)

#### 3.2.1. Within-Group Longitudinal Trajectories

Repeated-measures Analysis of Variance (RM-ANOVA) revealed statistically significant within-subject effects across the five assessment intervals (baseline, 1 month, 3 months, 6 months, and 12 months) for both treatment arms (Table 2).

In the MENS group, mean SPADI Pain scores decreased significantly from 70.29 ± 3.75 at baseline to 32.19 ± 3.21 at the 3-month milestone (*p* < 0.01). A further significant reduction was documented at 6 months (24.74 ± 2.36, *p* = 0.013), ultimately reaching 21.51 ± 2.09 at the 12-month endpoint. Although the trajectory between months 6 and 12 indicated continued clinical attenuation of pain (Figure 1), this terminal interval did not reach statistical significance (*p* = 0.090). Conversely, the TENS group showed an initial marked reduction from 66.00 ± 2.20 at baseline to 31.52 ± 3.66 at 3 months (*p* < 0.01), but subsequent evaluations demonstrated an upward rebound to 33.90 ± 3.10 at 6 months and 35.90 ± 2.82 at 12 months (*p* = 0.280).

**Figure 2:**
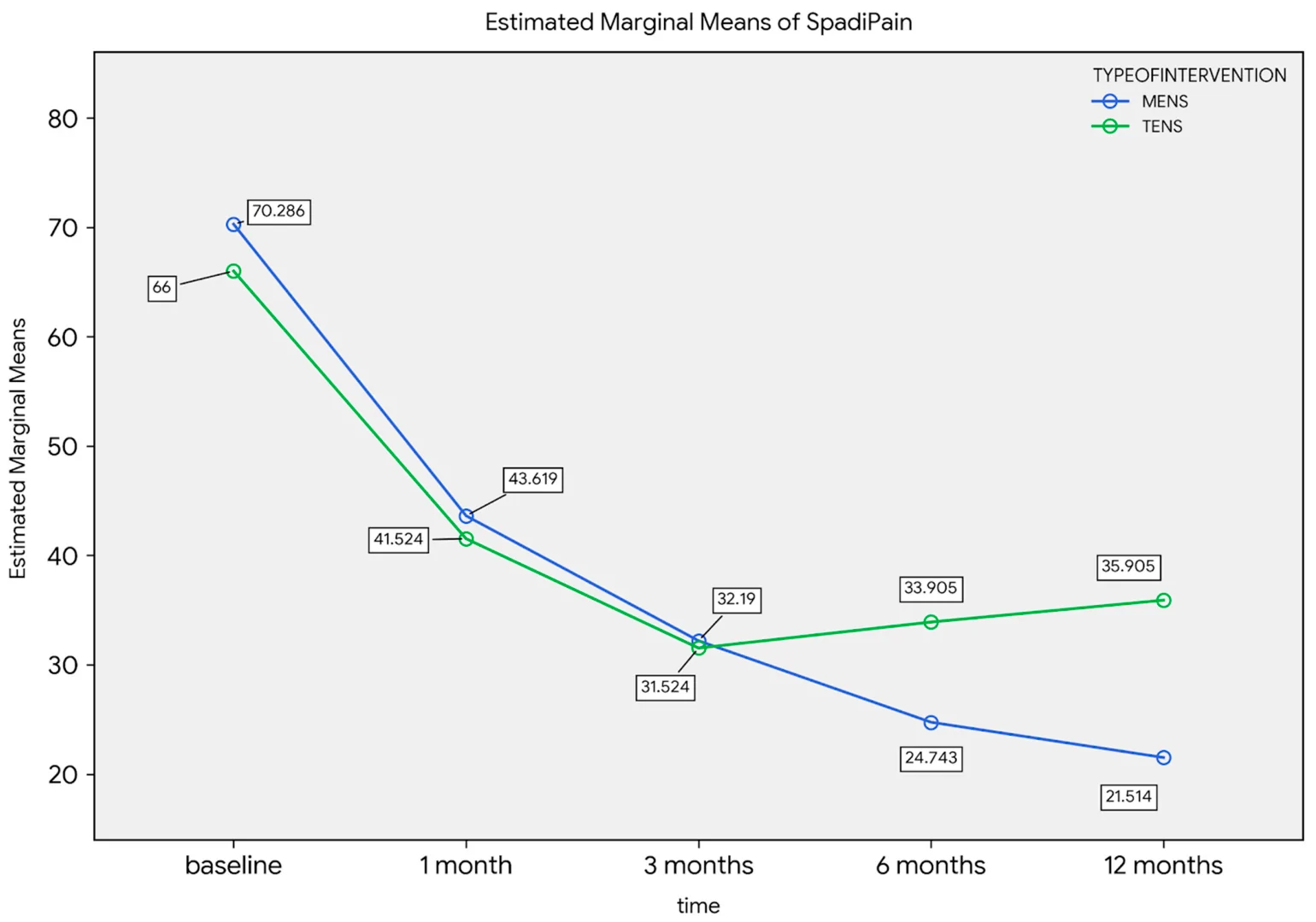
Mean values of SPADI Pain subscale outcomes at baseline 1, 3, 6 and 12 months follow up for each group. Significant difference between groups, at the 6 and 12 month follow up (*p*-values < 0.05).

For SPADI Disability scores, both cohorts demonstrated marked improvements at the 3-month mark, dropping from 49.94 ± 4.75 to 21.01 ± 3.12 in the MENS arm and from 52.20 ± 4.01 to 21.01 ± 2.94 in the TENS arm (*p* < 0.01 for both). Over the subsequent follow-up period, the MENS cohort sustained its functional recovery, recording a mean disability score of 19.89 ± 2.45 at 6 months and 18.24 ± 2.18 at 12 months (*p* > 0.05 relative to month 3). In stark contrast, the TENS cohort exhibited a statistically significant loss of functional recovery at 6 months (29.42 ± 1.99, *p* < 0.01), which deteriorated further by the 12-month follow-up (31.80 ± 2.09, *p* < 0.01; Figure 2).

#### 3.2.2. Between-Group Head-to-Head Comparisons

Two-way RM-ANOVA revealed a statistically significant “group” × “time” interaction for both SPADI Pain (F = 8.88, *p* = 0.001) and SPADI Disability (F = 5.27, *p* = 0.008). Independent-samples *t*-tests confirmed that while therapeutic efficacy was equivalent at post-intervention (1 month) and 3 months, outcomes diverged significantly at the mid- and long-term milestones (Table 3).

At 6 months, SPADI Pain was significantly lower in the MENS group compared to the TENS group, with a mean difference of 9.16 ± 3.89 (95% CI: 1.28 to 17.04, *p* = 0.024). This superiority broadened at the 12-month study endpoint, yielding a mean difference of 14.39 ± 3.51 (95% CI: 7.28 to 21.50, *p* < 0.001). Similarly, SPADI Disability was significantly lower in the MENS cohort at 6 months, with a mean difference of 9.53 ± 3.21 (95% CI: 3.04 to 16.03, *p* = 0.005), and at 12 months, demonstrating a between-group difference of 13.56 ± 2.89 (95% CI: 7.71 to 19.42, *p* < 0.001).

### 3.3. Non-Parametric Secondary Endpoints: NRS Pain and EQ-5D Health Status

Non-parametric longitudinal evaluations corroborated the parametric findings across all domains (Table 2 and Table 3).

#### 3.3.1. Numeric Rating Scale (NRS) for Pain

**Figure 3:**
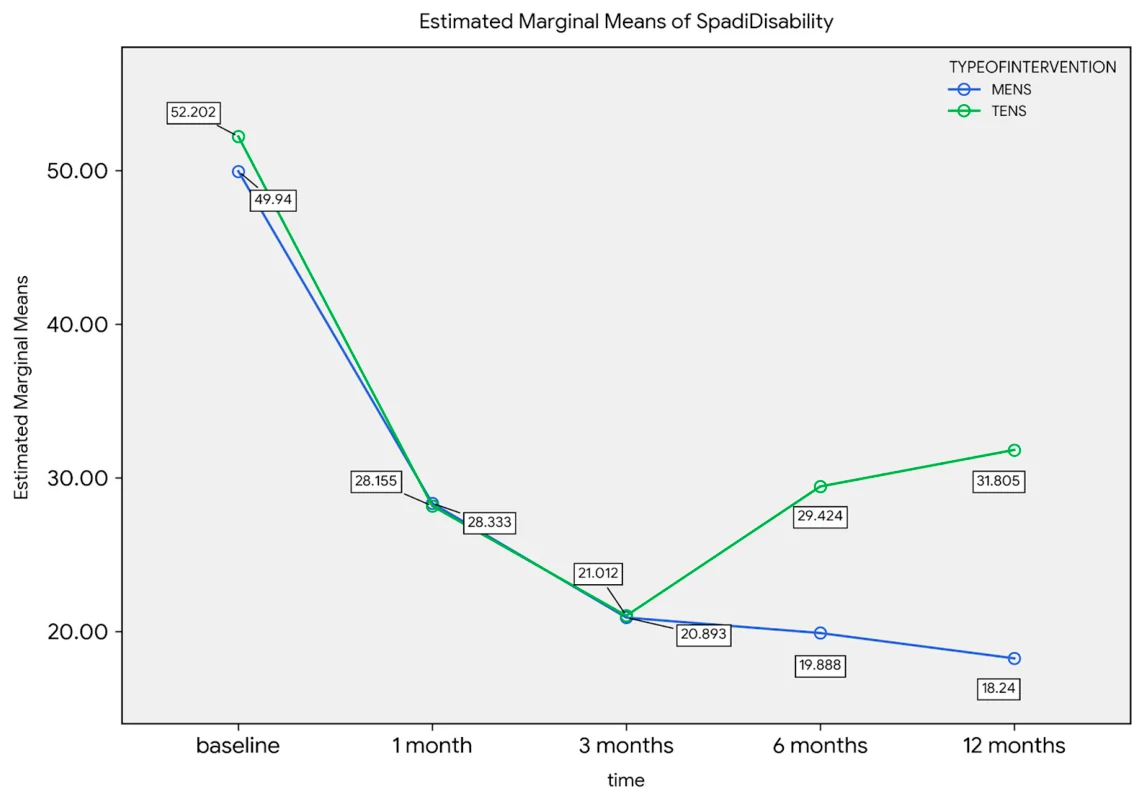
Mean values of SPADI Disability subscale outcomes at baseline 1, 3, 6 and 12 months follow up for each group. Significant difference between groups, at the 6 and 12 months follow up (*p*-values < 0.05).

Within the MENS group, Related-Samples Friedman analysis demonstrated continuous, statistically significant reductions in subjective pain intensity (*χ*^2^ = 78.42, *p* < 0.001). Mean NRS scores decreased from 6.52 ± 2.08 at baseline to 3.05 ± 1.80 at 3 months (*p* < 0.01), followed by a further significant decline to 1.19 ± 0.75 at 6 months and 0.86 ± 0.70 at the 12-month endpoint (*p* = 0.008; Figure 3). In the TENS cohort, pain scores decreased from 6.10 ± 1.90 at baseline to 2.71 ± 1.87 at 3 months (*p* < 0.01), maintained a comparable level at 6 months (2.52 ± 1.20, *p* = 0.717), but displayed a statistically significant relapse to 3.19 ± 1.25 by the 12-month follow-up (*p* < 0.01).

Between-group Mann–Whitney U comparisons showed that subjective pain was significantly lower in the MENS group at 6 months (mean difference: 1.33 ± 0.31, 95% CI: 0.70 to 1.96, *p* < 0.001) and at 12 months (mean difference: 2.33 ± 0.32, 95% CI: 1.68 to 2.99, *p* < 0.001).

#### 3.3.2. EuroQoL 5-Dimension (EQ-5D) Health Status

**Figure 4:**
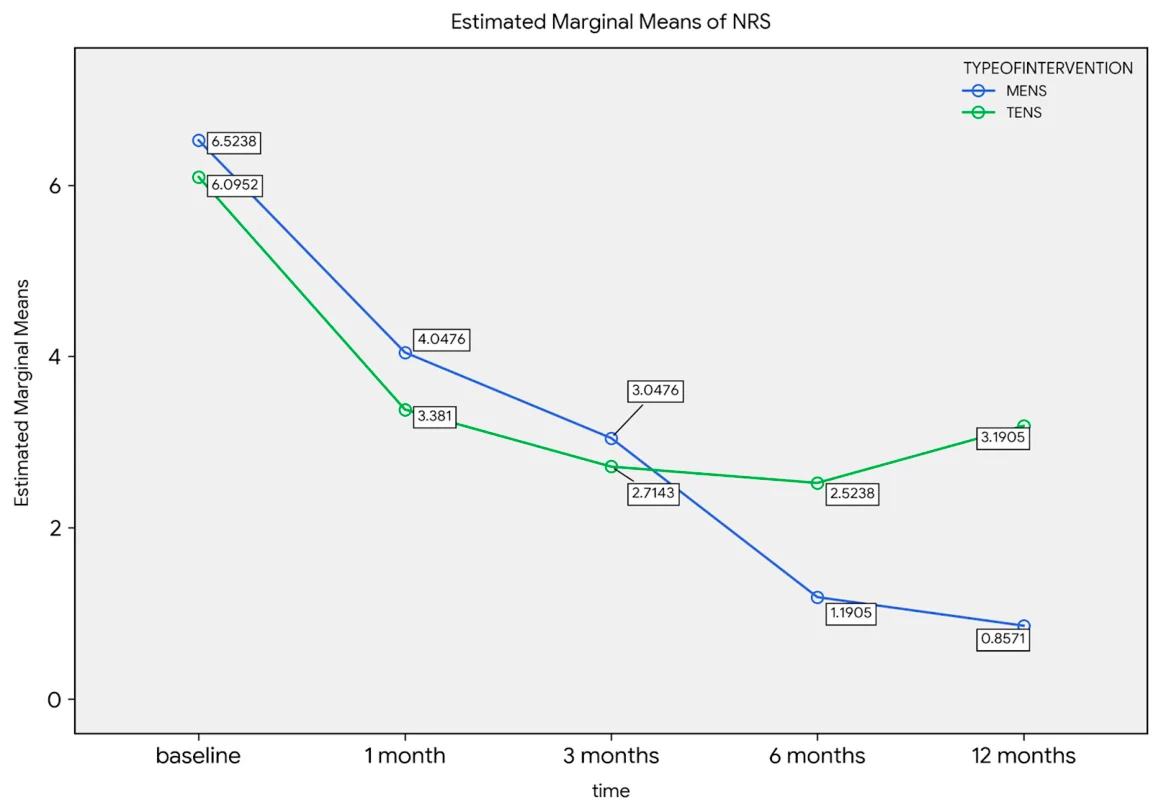
Mean values of Numeric Rating Scale (NRS) outcomes at baseline 1, 3, 6 and 12 months follow up for each group. Significant difference between groups, at the 6 and 12 months follow up (*p*-values < 0.05).

Both therapeutic protocols produced pronounced improvements in generic health-related quality of life during initial rehabilitation, with EQ-5D scores increasing from 70.95 ± 20.01 to 90.90 ± 8.80 in the MENS group (*p* < 0.01) and from 70.67 ± 16.48 to 93.67 ± 6.68 in the TENS group (*p* < 0.01) at the 3-month assessment (Figure 4).

**Figure 5:**
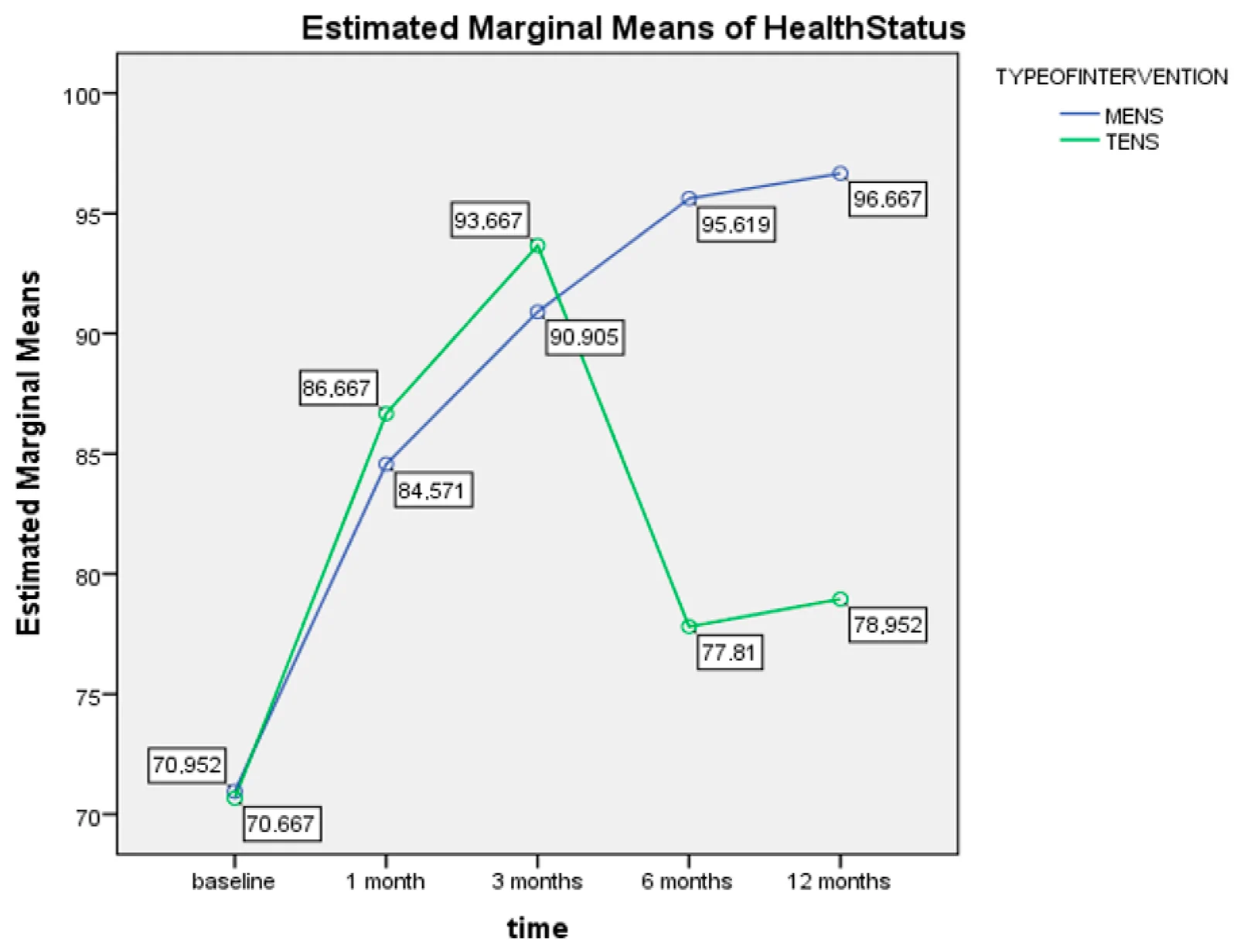
Mean values of (EQ 5-D) Health Status outcomes at baseline 1, 3, 6 and 12 months follow up for each group. Significant difference between groups, at the 6 and 12 months follow up (*p*-values < 0.05).

However, longitudinal stability differed markedly thereafter. The MENS cohort sustained these health status gains through month 12 (92.50 ± 5.10). In contrast, the TENS cohort demonstrated a statistically significant deterioration by month 6 (77.81 ± 5.60, *p* < 0.01), which persisted through the 12-month evaluation (74.79 ± 6.12). Between-group Mann–Whitney U testing established that health status scores were significantly higher in the MENS cohort at 6 months (mean difference: 17.81 ± 1.70, 95% CI: 14.35 to 21.27, *p* < 0.001) and at 12 months (mean difference: 17.71 ± 1.32, 95% CI: 15.05 to 20.38, *p* < 0.001) (Figure 5).

### 3.4. Structural Tear Remodeling via Musculoskeletal Ultrasound

Serial musculoskeletal ultrasonography identified distinct morphological patterns during the tissue remodelling phase between the 3-month and 12-month evaluations [56,57]. In the MENS arm, tear progression stabilized, exhibiting an incremental depth propagation of 0.02 mm and yielding a cumulative mean increase of 0.224 mm across the trial. Conversely, the TENS cohort demonstrated progressive structural enlargement, evidenced by an additional 0.45 mm expansion in tear depth over the same nine-month period, resulting in a cumulative mean deterioration of 0.66 mm at the 12-month endpoint.

## 4. Discussion

### 4.1. Clinical Efficacy of Conservative Management and Multimodal Synergy

Conservative management represents the clinical cornerstone for partial-thickness rotator cuff tears, offering a cost-effective, low-morbidity intervention that addresses functional deficits alongside psychological barriers such as kinesiophobia and pain-related distress [7,8]. Although surgical repair is frequently considered when structural discontinuity exceeds 50% of tendon thickness [5], contemporary clinical trials demonstrate that structured physical rehabilitation yields medium- and long-term functional recovery equivalent to operative repair in middle-aged cohorts [2,19]. This equivalence reinforces the concept that clinical recovery in rotator cuff disease is predominantly governed by adaptive neuromuscular compensation, load tolerance restoration, and tissue-level mechanotransduction rather than absolute macroscopic anatomical restitution [17,18].

A vital methodological consideration in interpreting our findings is that both treatment cohorts completed an identical, supervised kinesiotherapy regimen alongside their assigned electrophysical protocol. Therefore, the observed therapeutic divergences do not reflect the isolated efficacy of MENS or TENS as standalone monotherapies, but rather their distinct additive or synergistic contributions when integrated into an active exercise baseline [16,58]. Therapeutic exercise remains the essential engine of conservative care, stimulating tenocyte metabolism and matrix realignment via mechanotransductive pathways [8,17]. Within this multimodal framework, electrophysical modalities serve as adjunctive interventions [16,59]. Our findings demonstrate that while both modalities facilitated acute exercise engagement, their long-term biological interactions with exercise diverged fundamentally.

Disentangling the therapeutic contributions of the overarching rehabilitation program from the specific adjunctive effects of electrical stimulation is essential for valid clinical interpretation. Structured therapeutic exercise remains the undisputed cornerstone of conservative management for rotator cuff tears, driving mechanotransductive tissue remodeling, restoring scapulothoracic kinematics, and rebuilding muscular strength. The substantial, parallel improvements observed in both cohorts immediately post-intervention (1 month) largely reflect the efficacy of this core kinesiotherapy regimen, facilitated by the acute pain-relieving coverage of both electrophysical modalities. The distinct adjunctive effect of MENS versus TENS emerged solely during the long-term, post-intervention phase (3 to 12 months). While conventional TENS acted as a transient neurosensory analgesic that waned following cessation, MENS provided a sustained biological adjuvant—evidenced by the long-term preservation of functional scores and sonographic tear containment. Thus, MENS did not replace active rehabilitation but acted synergistically as a cellular modifier that consolidated and preserved the structural and functional gains achieved through exercise therapy.

### 4.2. Longitudinal Divergence: TENS vs. MENS Efficacy and Clinical Significance

A pivotal finding of this randomized trial is the striking divergence in therapeutic longevity between cohorts over the 12-month monitoring window. Within the acute post-treatment phase (up to 3 months), both MENS and TENS elicited robust, statistically equivalent reductions in SPADI pain (approximately 54%) and functional disability (approximately 58%), accompanied by parallel improvements in generic EQ-5D health status [28,29]. However, their post-intervention trajectories diverged substantially thereafter. The MENS cohort demonstrated sustained and continuing functional recovery through months 6 and 12, whereas the TENS cohort experienced progressive loss of initial gains, marked by symptom recrudescence and functional decline.

Beyond formal statistical significance (*p* < 0.01), this divergence represents a genuine, patient-perceived functional advantage when benchmarked against established Minimal Clinically Important Difference (MCID) thresholds. In rotator cuff pathologies, the accepted MCID for the SPADI ranges from 8.0 to 13.2 points [60], while a change of 1.5 to 2.0 points constitutes the clinical threshold for the NRS pain scale [61]. In the present study, between-group differences at month 6 approached or fulfilled the lower MCID boundary (SPADI Pain: 9.16 points; SPADI Disability: 9.53 points). By the primary 12-month endpoint, the inter-group divergence substantially exceeded conservative MCID limits, reaching 14.39 points (95% CI: 7.28 to 21.50) for SPADI Pain and 13.56 points (95% CI: 7.71 to 19.42) for SPADI Disability. Concurrently, the 12-month between-group NRS divergence expanded to 2.33 points (95% CI: 1.68 to 2.99), surpassing the 2.0-point threshold for meaningful clinical analgesia. These metrics verify that the integration of MENS provided lasting, tangible symptom stabilization, shielding patients from the functional regression documented following TENS cessation.

### 4.3. Neurobiological and Cytological Mechanisms of Microcurrent Therapy

The extended therapeutic longevity achieved with MENS appears fundamentally rooted in its cytological, bioelectric, and trophic mode of action. Delivered at subsensory micro-amperage intensities, MENS avoids the neural accommodation and discomfort characteristic of millicurrent modalities, coupling directly with endogenous bioelectric tissue fields [23,24]. At the cellular level, micro-amperage stimulation functions as an energetic catalyst, driving mitochondrial ATP resynthesis up to fivefold while facilitating amino acid transport, membrane transport, and protein synthesis [23]. Beyond peripheral bioenergetics, contemporary evidence indicates that microcurrent stimulation modulates chronic central nociceptive transmission via hypothalamic pathways and autonomic recalibration [23].

Biochemically, microcurrent fields orchestrate the transition from chronic catabolic inflammation toward constructive extracellular matrix remodeling. Microcurrent stimulation attenuates pro-inflammatory mediators (such as TNF-α, IL-1β, and IL-6) while upregulating anabolic factors, including TGF-β and VEGF, which accelerate neo-vascularization, enhance tenocyte cellularity, and drive oriented type I collagen synthesis [62,63]. These cytological mechanisms are consistent with the experimental findings of Jonik et al. [64], who demonstrated superior structural reconstitution, mechanical load tolerance, and collagen alignment in animal models of rotator cuff tears treated with microcurrent regimens.

At the level of clinical evidence, pooled findings from systematic reviews corroborate the utility of microcurrent applications in upper-limb tendinopathies. Iijima and Takahashi [65] confirmed that microcurrent therapy delivers reliable pain relief and functional restoration in musculoskeletal conditions, noting that its subsensory profile promotes high patient compliance [66]. In their broad evaluation of conservative options for rotator cuff-related shoulder pain, Johnson et al. [59] highlighted microcurrent therapy as one of the few modalities capable of sustaining intermediate- and long-term functional recovery, outperforming sensory-level currents. Furthermore, systematic evidence by Alahmari et al. [26] revealed that pairing microcurrent interventions with therapeutic exercise produces significantly superior improvements in pain-free abduction and isometric cuff strength compared to exercise alone or exercise paired with TENS. Our findings align closely with Poltawski and Watson [24], who reported lasting clinical benefits extending across 3, 6, and 12 months following microcurrent therapy in chronic recalcitrant tendinopathies.

### 4.4. Neurophysiological Limitations of TENS in Long-Term Tendon Rehabilitation

In sharp contrast, conventional TENS functions predominantly as an immediate, symptom-modulating neural filter rather than an anabolic stimulus [67,68]. The neurobiological action of TENS depends on the Melzack–Wall spinal gate mechanism—wherein high-frequency currents selectively excite large-diameter, myelinated Aβcutaneous afferents to segmentally inhibit nociceptive Aδ and C projection neurons in the dorsal horn—or on low-frequency stimulation that triggers endogenous opioid release across descending bulbospinal pathways [21,27,69]. While repeated applications momentarily attenuate secondary hyperalgesia and downregulate hyperexcitable central neurons [22], TENS exerts negligible direct regenerative or trophic influence upon damaged tendon architecture [16,69].

Systematic reviews in rotator cuff disease consistently confirm this transient efficacy profile. A comprehensive Cochrane review by Page et al. [16] concluded that while TENS provides clinically appreciable short-term analgesia superior to placebo during the active treatment block, these benefits fail to persist at intermediate and long-term follow-up intervals. This post-cessation decay was corroborated by Desjardins-Charbonneau, A. et al. [69], who determined that passive modalities like TENS offer limited supplementary value when added to exercise beyond the immediate intervention window, showing no meaningful preservation of shoulder function, active elevation range, or quality of life at 6 to 12 months. Recent network meta-analyses reinforce these boundaries: Johnson et al. [59] noted that while TENS exhibits favorable effect sizes for acute pain (<6 weeks), its clinical efficacy regresses steeply by month 3, demonstrating no superiority over exercise or sham controls at 6 and 12 months. Broad reviews by Steuri et al. [70] and Pieters et al. [58] affirmed that passive electrotherapy modalities do not confer additive value to long-term recovery, structural adaptation, or persistent disability reduction.

Because TENS does not restore cellular bioenergetics, stimulate tenocyte proliferation, or reverse local insertional hypovascularity, its analgesic benefits systematically erode once stimulation ceases. This mechanistic ceiling explains our longitudinal results: while TENS provided rapid functional relief that facilitated active exercise in the acute phase (up to Month 3), it was unable to maintain tissue load tolerance over time, culminating in symptomatic recrudescence and disability escalation at months 6 and 12.

### 4.5. Musculoskeletal Ultrasound and the Clinicoradiological Paradox

Serial musculoskeletal ultrasonography highlighted the recognized clinicoradiological paradox: the discordance between morphological tissue state and subjective clinical outcomes [71]. Between baseline and month 3, both cohorts demonstrated a comparable increase in mean tear depth of approximately 0.20 mm (*p* < 0.05). However, marked morphological divergence occurred during the remodeling window between months 3 and 12. In the MENS cohort, tear progression largely stabilized, exhibiting a minimal secondary increase of 0.02 mm (cumulative mean propagation: 0.224 mm). Conversely, the TENS cohort demonstrated structural enlargement, marked by an additional 0.45 mm propagation over the identical nine-month period (cumulative mean deterioration: 0.66 mm). Despite structural progression in both groups, all patients maintained significant functional and pain improvements compared to baseline. This phenomenon mirrors findings by Sambandam et al. [71] who emphasized that structural tear expansion does not systematically dictate clinical failure. In patients aged over 50 years, local microvascular hypovascularity at the insertion is common, and systemic compounding factors—such as age, mechanical shear stress, and thoracic posture—exert continuous degenerative pressures on tendon morphology [5,6]. Nevertheless, the substantial stabilization of tear depth observed in the MENS cohort suggests that microcurrent fields, combined with mechanical loading, facilitated local collagen stability and constrained longitudinal tear propagation, corroborating the structural outcomes reported by Tsuchiya et al. [57].

### 4.6. Strengths, Limitations, and Practical Recommendations

Methodological strengths of this clinical trial include its prospective, randomized, participant- and outcome assessor-blinded design, adherence to CONSORT reporting criteria, and complete 100% participant retention across the 12-month monitoring window, eliminating attrition bias [30,32]. Additionally, patient recruitment across two specialized centers provided a homogenous baseline [29].

Several limitations of this study should be acknowledged. First, the relatively small sample size (N = 42) limits the statistical power, particularly for secondary outcomes and multiple time-point comparisons, increasing the potential for type II error. Although an a priori power analysis justified this sample size for detecting primary interaction effects, future large-scale, multicenter randomized controlled trials are necessary to validate our findings. Second, because both study arms received an identical, evidence-based supervised kinesiotherapy regimen, the observed therapeutic gains reflect the synergistic and adjunctive contribution of MENS rather than its performance as an isolated monotherapy. Consequently, conclusions regarding the superiority of MENS should be interpreted within the context of multimodal conservative management.

Bilateral Involvement: In the single participant (n = 1, 2.38%) presenting with bilateral symptoms, intervention and outcome tracking were restricted exclusively to the primary symptomatic index shoulder, preventing unit-of-analysis bias but leaving contralateral influences unmeasured.

Co-interventions During Follow-Up: We acknowledge the potential confounding influence of sporadic, self-administered over-the-counter analgesics or unrecorded lifestyle modifications across the 12-month timeline.

Ultrasound Measurement Variability: We address the inherent operator dependency of musculoskeletal ultrasound, noting that despite standardized examination by a single blinded expert [56], minor millimeter-level technical measurement variations remain a recognized limitation.

#### Practical Recommendations for Clinical Practice

From a clinical standpoint, practitioners must distinguish between immediate pain-gating and durable tissue stabilization. Although TENS serves as an effective, low-cost analgesic bridge to facilitate early exercise compliance, its therapeutic benefits remain strictly transient. In contrast, integrating subsensory MENS (e.g., 100 μA, 50 Hz) provides prolonged functional recovery, containment of tear progression, and cellular bioenergetic support without inducing neuromuscular fatigue. Consequently, physical therapists should prioritize microcurrent therapy when sustained structural and functional preservation is targeted, while extending clinical surveillance beyond standard 6- to 12-week discharge timelines to accurately detect late-stage symptom recurrence.

## 5. Conclusions

In conclusion, when integrated into a structured kinesiotherapy regimen for partial-thickness rotator cuff tears, subsensory MENS appears to provide more durable long-term symptomatic and functional stabilization compared to conventional TENS. However, given the modest sample size and the concurrent application of therapeutic exercise in both cohorts, these findings should be interpreted with caution. MENS should be regarded as a promising adjunctive modality whose long-term disease-modifying potential warrants further validation in larger multicenter cohorts.

## Figures and Tables

**Table 1:** Baseline Demographic and Clinical Characteristics Of the Study Participants.

Baseline Characteristic	Total Cohort (*n* = 42)	MENS Group (*n* = 21)	TENS Group (*n* = 21)	*p*-Value
Age (years), Mean ± SD	46.71 ± 12.24	47.10 ± 12.55	46.33 ± 12.22	0.841
Gender (Male/Female), *n* (%)	22 (52.4%)/20 (47.6%)	11 (52.4%)/10 (47.6%)	11 (52.4%)/10 (47.6%)	1.000
Dominant Hand (Right/Left), *n* (%)	38 (90.5%)/4 (9.5%)	19 (90.5%)/2 (9.5%)	19 (90.5%)/2 (9.5%)	1.000
Affected Shoulder (Right/Left), *n* (%)	25 (59.5%)/17 (40.5%)	13 (61.9%)/8 (38.1%)	12 (57.1%)/9 (42.9%)	0.753
Involvement of Dominant Arm, *n* (%)	29 (69.0%)	15 (71.4%)	14 (66.7%)	0.741
Occupational Physical Demand, *n* (%)				0.942
• Sedentary/Office work	17 (40.5%)	9 (42.9%)	8 (38.1%)	
• Light-to-Moderate physical work	17 (40.5%)	8 (38.1%)	9 (42.9%)	
• Heavy manual labor/Overhead load	8 (19.0%)	4 (19.0%)	4 (19.0%)	
Tegner Activity Scale, Median (IQR)	3.0 (2.0–4.0)	3.0 (2.0–4.0)	3.0 (2.0–4.0)	0.884
Recreational Overhead Sports, *n* (%)	11 (26.2%)	6 (28.6%)	5 (23.8%)	0.725
Baseline SPADI Pain (0–100), Mean ± SD	68.14 ± 3.82	70.29 ± 3.75	66.00 ± 2.20	0.334
Baseline SPADI Disability (0–100), Mean ± SD	51.07 ± 4.41	49.94 ± 4.75	52.20 ± 4.01	0.718
Baseline NRS Pain (0–10), Mean ± SD	6.31 ± 1.98	6.52 ± 2.08	6.10 ± 1.90	0.505
Baseline EQ-5D Health Status, Mean ± SD	70.81 ± 18.11	70.95 ± 20.01	70.67 ± 16.48	0.961

*Note: Values are Mean ± SD, n (%), or Median (IQR). En dashes (–) denote ranges. SPADI: Shoulder Pain and Disability Index; NRS: Numerical Rating Scale; EQ-5D: EuroQol 5-Dimension; MENS: Microcurrent Electrical Neuromuscular Stimulation; TENS: Transcutaneous Electrical Nerve Stimulation.*

**Table 2:** Longitudinal Clinical Outcomes Across Follow-up Timepoints.

Outcome Measure/Study Arm	Baseline (T0)	1 Month (T1)	3 Months (T2)	6 Months (T3)	12 Months (T4)	Within-Group *p*-Value
SPADI Pain (0–50)						
—MENS Group (*n* = 21)	70.29 ± 3.75	44.10 ± 3.50	32.19 ± 3.21	24.74 ± 2.36	21.51 ± 2.09	<0.001 ^a^
—TENS Group (*n* = 21)	66.00 ± 2.20	43.80 ± 3.10	31.52 ± 3.66	33.90 ± 3.10	35.90 ± 2.82	<0.001 ^a^
SPADI Disability (0–80)						
—MENS Group (*n* = 21)	49.94 ± 4.75	31.40 ± 3.80	21.01 ± 3.12	19.89 ± 2.45	18.24 ± 2.18	<0.001 ^a^
—TENS Group (*n* = 21)	52.20 ± 4.01	32.10 ± 3.50	21.01 ± 2.94	29.42 ± 1.99	31.80 ± 2.09	<0.001 ^a^
Numeric Rating Scale (NRS, 0–10)						
—MENS Group (*n* = 21)	6.52 ± 2.08	4.20 ± 1.40	3.05 ± 1.80	1.19 ± 0.75	0.86 ± 0.70	<0.001 ^b^
—TENS Group (*n* = 21)	6.10 ± 1.90	4.10 ± 1.30	2.71 ± 1.87	2.52 ± 1.20	3.19 ± 1.25	<0.001 ^b^
EQ-5D Health Status (0–100)						
—MENS Group (*n* = 21)	70.95 ± 20.01	82.40 ± 12.10	90.90 ± 8.80	92.10 ± 6.40	92.50 ± 5.10	<0.001 ^b^
—TENS Group (*n* = 21)	70.67 ± 16.48	83.10 ± 11.50	93.67 ± 6.68	77.81 ± 5.60	74.79 ± 6.12	<0.001 ^b^

Values are expressed as Mean ± SD. Abbreviations: SPADI, Shoulder Pain and Disability Index; NRS, Numeric Rating Scale; EQ-5D, EuroQoL 5-Dimension; MENS, Microcurrent Electrical Neuromuscular Stimulation; TENS, Transcutaneous Electrical Nerve Stimulation. ^a^ Repeated Measures ANOVA within-subject effect across time (*p* < 0.05). ^b^ Related-Samples Friedman Two-Way ANOVA by ranks (*p* < 0.05).

**Table 3:** Between-Group Comparison Of Clinical Outcomes Across Follow-up Time Points.

Outcome Measure/Time Point	MENS (*n* = 21)	TENS (*n* = 21)	Mean Diff. ± SE	95% Confidence Interval	Test Statistic	*p*-Value
SPADI Pain (0–50)						
—Baseline (T0)	70.29 ± 3.75	66.00 ± 2.20	4.29 ± 0.95	−1.15 to 9.73	*t* = 1.06	0.295
—1 Month (T1)	44.10 ± 3.50	43.80 ± 3.10	0.30 ± 1.02	−1.76 to 2.36	*t* = 0.29	0.770
—3 Months (T2)	32.19 ± 3.21	31.52 ± 3.66	0.67 ± 1.06	−1.48 to 2.82	*t* = 0.63	0.532
—6 Months (T3)	24.74 ± 2.36	33.90 ± 3.10	9.16 ± 3.89	1.28 to 17.04	*t* = 2.35	0.024 *
—12 Months (T4)	21.51 ± 2.09	35.90 ± 2.82	14.39 ± 3.51	7.28 to 21.50	*t* = 4.10	<0.001 *
SPADI Disability (0–80)						
—Baseline (T0)	49.94 ± 4.75	52.20 ± 4.01	−2.26 ± 1.35	−4.99 to 0.47	*t* = −0.40	0.690
—1 Month (T1)	31.40 ± 3.80	32.10 ± 3.50	−0.70 ± 1.13	−2.98 to 1.58	*t* = −0.62	0.539
—3 Months (T2)	21.01 ± 3.12	21.01 ± 2.94	0.00 ± 0.94	−1.89 to 1.89	*t* = 0.00	1.000
—6 Months (T3)	19.89 ± 2.45	29.42 ± 1.99	9.53 ± 3.21	3.04 to 16.03	*t* = 2.97	0.005 *
—12 Months (T4)	18.24 ± 2.18	31.80 ± 2.09	13.56 ± 2.89	7.71 to 19.42	*t* = 4.69	<0.001 *
Numeric Rating Scale (NRS, 0–10)						
—Baseline (T0)	6.52 ± 2.08	6.10 ± 1.90	0.42 ± 0.61	−0.82 to 1.66	*U* = 201.5	0.505
—1 Month (T1)	4.20 ± 1.40	4.10 ± 1.30	0.10 ± 0.42	−0.75 to 0.95	*U* = 212.0	0.812
—3 Months (T2)	3.05 ± 1.80	2.71 ± 1.87	0.34 ± 0.57	−0.81 to 1.49	*U* = 205.0	0.650
—6 Months (T3)	1.19 ± 0.75	2.52 ± 1.20	1.33 ± 0.31	0.70 to 1.96	*U* = 88.5	<0.001 *
—12 Months (T4)	0.86 ± 0.70	3.19 ± 1.25	2.33 ± 0.32	1.68 to 2.99	*U* = 35.0	<0.001 *
EQ-5D Health Status (0–100)						
—Baseline (T0)	70.95 ± 20.01	70.67 ± 16.48	0.28 ± 5.66	−11.16 to 11.72	*U* = 219.0	0.961
—1 Month (T1)	82.40 ± 12.10	83.10 ± 11.50	−0.70 ± 3.64	−8.06 to 6.66	*U* = 214.5	0.865
—3 Months (T2)	90.90 ± 8.80	93.67 ± 6.68	−2.77 ± 2.41	−7.64 to 2.10	*U* = 195.0	0.485
—6 Months (T3)	92.10 ± 6.40	77.81 ± 5.60	17.81 ± 1.70	14.35 to 21.27	*U* = 32.0	<0.001 *
—12 Months (T4)	92.50 ± 5.10	74.79 ± 6.12	17.71 ± 1.32	15.05 to 20.38	*U* = 28.5	<0.001 *

Values for MENS and TENS are presented as Mean ± SD. Abbreviations: SE, Standard Error; CI, Confidence Interval; SPADI, Shoulder Pain and Disability Index; NRS, Numeric Rating Scale; EQ-5D, EuroQoL 5-Dimension; *t*, Independent-samples *t*-test statistic; *U*, Mann-Whitney *U* test statistic. * Statistically significant between-group difference (*p* < 0.05).

## Data Availability

The data presented in this study are available upon request from the corresponding author. The data are not publicly available due to the applicable data protection law in Greece (Law 4624/2019).

## Abbreviations

The following abbreviations are used in this manuscript:

TENS	Transcutaneous Electrical Nerve Stimulation
MENS	Microcurrent Electrical Neuromuscular Stimulation
SPADI	Shoulder Pain and Disability Index
NRS	Numeric Rating Scale
EQ	EuroQol 5-Dimension Questionnaire
